# Effects of Dietary Nonfibrous Carbohydrate/Neutral Detergent Fiber Ratio on Methanogenic Archaea and Cellulose-Degrading Bacteria in the Rumen of Karakul Sheep: a 16S rRNA Gene Sequencing Study

**DOI:** 10.1128/aem.01291-22

**Published:** 2022-12-21

**Authors:** Tiantian Bai, Xuanxuan Pu, Xuefeng Guo, Junfeng Liu, Linbo Zhao, Xiuping Zhang, Sujiang Zhang, Long Cheng

**Affiliations:** a College of Animal Science and Technology, Tarim University, Alar, Xinjiang, People’s Republic of China; b Key Laboratory of Tarim Animal Husbandry Science and Technology of Xinjiang Production and Construction Group, Alar, Xinjiang, People’s Republic of China; c Faculty of Veterinary and Agricultural Sciences, Dookie College, The University of Melbourne, Melbourne, Victoria, Australia; University of Nebraska—Lincoln

**Keywords:** Karakul sheep, 16S rRNA gene sequencing, cellulose-degrading bacteria, fiber, methane

## Abstract

The study was conducted to investigate the effects of dietary nonfibrous carbohydrate (NFC)/neutral detergent fiber (NDF) ratio on methanogenic archaea and cellulose-degrading bacteria in Karakul sheep by 16S rRNA gene sequencing. Twelve Karakul sheep were randomly divided into four groups, each group with three replicates, and they were fed with four dietary NFC/NDF ratios at 0.54, 0.96, 1.37, and 1.90 as groups 1, 2, 3, and 4, respectively. The experiment lasted for four periods: I (1 to 18 days), II (19 to 36 days), III (37 to 54 days), and IV (55 to 72 days); during each period, rumen contents were collected before morning feeding to investigate on methanogenic archaea and cellulose-degrading bacteria. The results showed that with an increase in dietary NFC/NDF ratio, the number of rumen archaea operational taxonomic units and the diversity of archaea decrease. The most dominant methanogens did not change with dietary NFC/NDF ratio and prolongation of experimental periods. *Methanobrevibacter* was the most dominant genus. At the species level, the relative abundance of *Methanobrevibacter ruminantium* first increased and then decreased when the NFC/NDF ratio increased. When the dietary NFC/NDF ratio was 0.96, the structure of archaea was largely changed, and the relative abundance of *Fibrobacter* sp. strain UWCM, *Ruminococcus flavefaciens*, and *Ruminococcus albus* were the highest. When the dietary NFC/NDF ratio was 1.37, the relative abundance of *Butyrivibrio fibrisolvens* was higher than for other groups. Based on all the data, we concluded that a dietary NFC/NDF ratio of ca. 0.96 to 1.37 was a suitable ratio to support optimal sheep production.

**IMPORTANCE** CH_4_ produced by ruminants aggravates the greenhouse effect and cause wastage of feed energy, and CH_4_ emissions are related to methanogens. According to the current literature, there is a symbiotic relationship between methanogens and cellulolytic bacteria, so reducing methane will inevitably affect the degradation of fiber materials. This experiment used 16S rRNA gene high-throughput sequencing technology to explore the balance relationship between methanogens and cellulolytic bacteria for the first time through a long-term feeding period. The findings provide fundamental data, supporting for the diet structures with potential to reduce CH_4_ emission.

## INTRODUCTION

The Intergovernmental Panel on Climate Change showed that greenhouse effects of methane (CH_4_) is 21-fold greater than carbon dioxide (CO_2_) and 300-fold greater than nitrous oxide (N_2_O) (1). Based on data from the U.S. Environmental Protection Agency (2), the direct livestock contribution to CH_4_ and N_2_O emissions can be estimated at 7.5% of the global greenhouse gas emission for 2020. The CH_4_ emission from ruminants has become one of the three major CH_4_ emission sources, and it has attracted the attention of many countries for research and mitigation (3, 4). Apart from polluting the environment, the production of CH_4_ also causes ruminant feed energy loss (5, 6).

The development of technology such as high-throughput method (7) makes it possible to study the details of rumen microbiome. 16S rRNA gene amplification sequencing is one of them, it has become an important means to study the composition and structure of microbial communities in environmental samples (8–10). The rumen microbiota is mainly composed of bacteria, eukaryotes, protozoa, and archaea (11, 12), and methanogens and cellulolytic bacteria have a mutually beneficial symbiotic relationship in the rumen. The vast majority of fiber-degrading bacteria are hydrogen-producing bacteria (13), and the methanogenesis process is also closely related to the activity of fiber-degrading bacteria. Many studies have shown that diet structure can affect the viability of methanogens and fiber-degrading bacteria. Studies have shown that high-carbohydrate diets reduce the activity of methanogens (14) and the number of fiber-degrading bacteria (15), resulting in decreased fiber digestibility and increased risk of acidosis (16). This is mainly because when the concentration of fiber in the diet is high, the rumen undergoes propionic acid fermentation, which consumes hydrogen (17) and reduces CH_4_ emissions. It is interesting to note that, at the same time, the carbohydrate fermentation produces volatile fatty acid (VFA), which reduces the rumen pH, causing hydrogen accumulation and ultimately inhibiting fiber degradation (18, 19). When the fiber content of the diet is high, the rumen undergoes acetic and butyric fermentations, producing acetic and butyric acids and hydrogen (20), which are then utilized by methanogens to synthesize CH_4_. Therefore, while formulating the diet structure to reduce CH_4_ emission, we should also study the changes in rumen fiber-degrading bacteria and fiber-degrading rate in the diet. Nonfibrous carbohydrate (NFC) in the Cornell Net Carbohydrate and Protein System was used to design this experimental diet, which comprehensively represented the digestible carbohydrate part of the diet. Neutral detergent fiber (NDF) refers to the part of the diet that is insoluble in neutral detergent, mainly representing the content of fiber in the diet. The dietary NFC/NDF ratio is a good indicator to represent the ratio of digestible carbohydrates to fiber in the diet. Moreover, the rumen microbiota is very complex and can change over time. Therefore, in this experiment, sheep were fed with four NFC/NDF ratios diets for 72 days, and a 16S rRNA gene high-throughput sequencing technique was used to determine the flora structure of methanogens and cellulose-degrading bacteria in the four stages of the entire experimental period.

## RESULTS

### Number of archaea OTUs.

**FIG 1:**
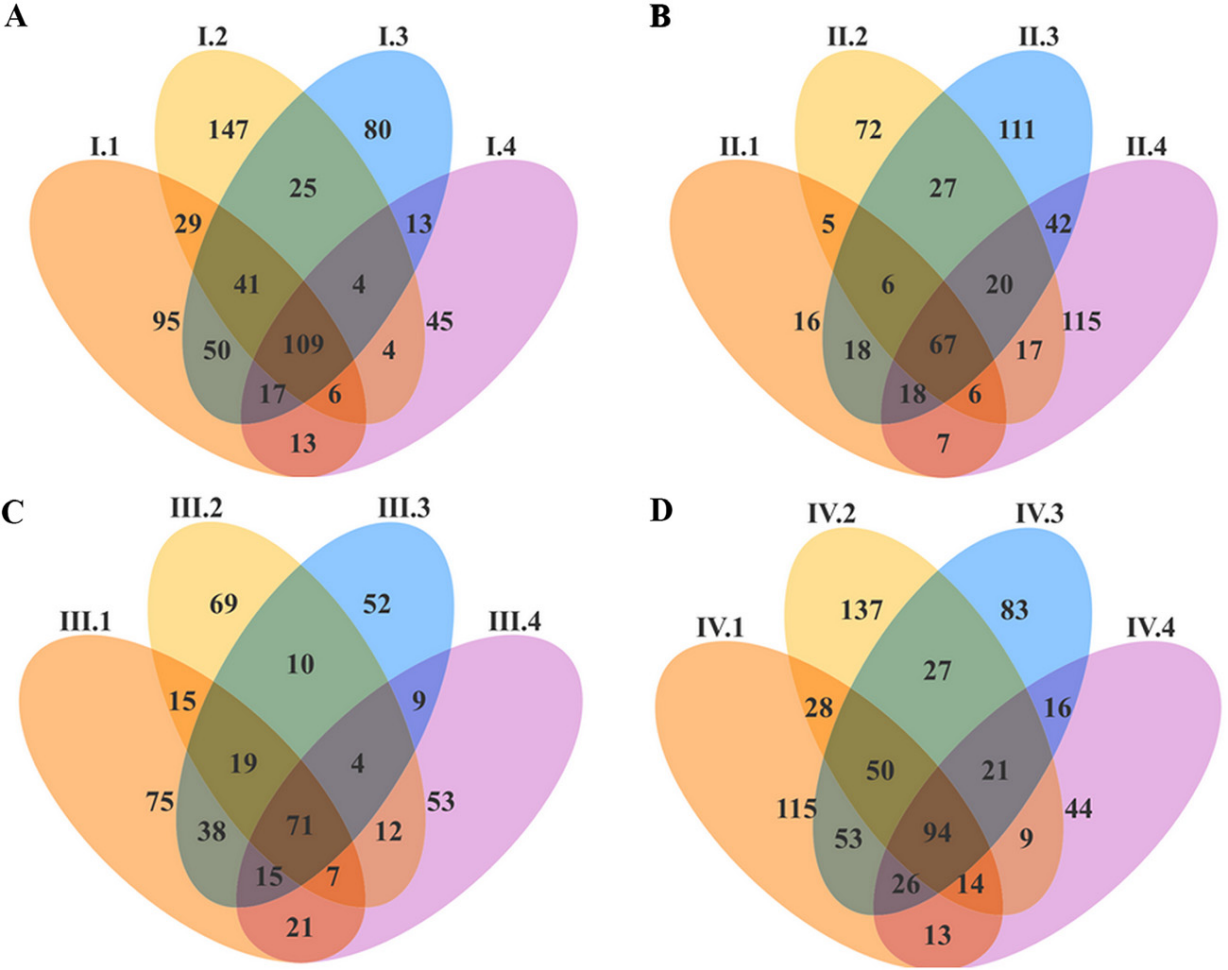
Venn graph of archaea OTUs in Karakul sheep. (A, B, C, and D) Periods I, II, III, and IV, respectively. Periods I (1 to 18 days), II (19 to 36 days), III (37 to 54 days), and IV (55 to 72 days) and groups 1, 2, 3, and 4 represent four groups of Karakul sheep treated with four dietary NFC/NDF ratios of 0.54, 0.96, 1.37, and 1.90, respectively. The same color represents the same OTUs among each group, while different colors represent the unique OTUs of each group in the figure (as in subsequent figures).

A Venn graph was drawn using the results of annotated OTUs and by analyzing common and unique OTUs among samples for four periods. As shown in Fig. 1, the numbers of OTUs among the four periods were as follows: group 1 > group 2 > group 3 > group 4. Groups 1 to 4 reflect four groups of Karakul sheep with four dietary NFC/NDF ratios of 0.54, 0.96, 1.37, and 1.90, respectively.

### Rarefaction curve.

**FIG 2:**
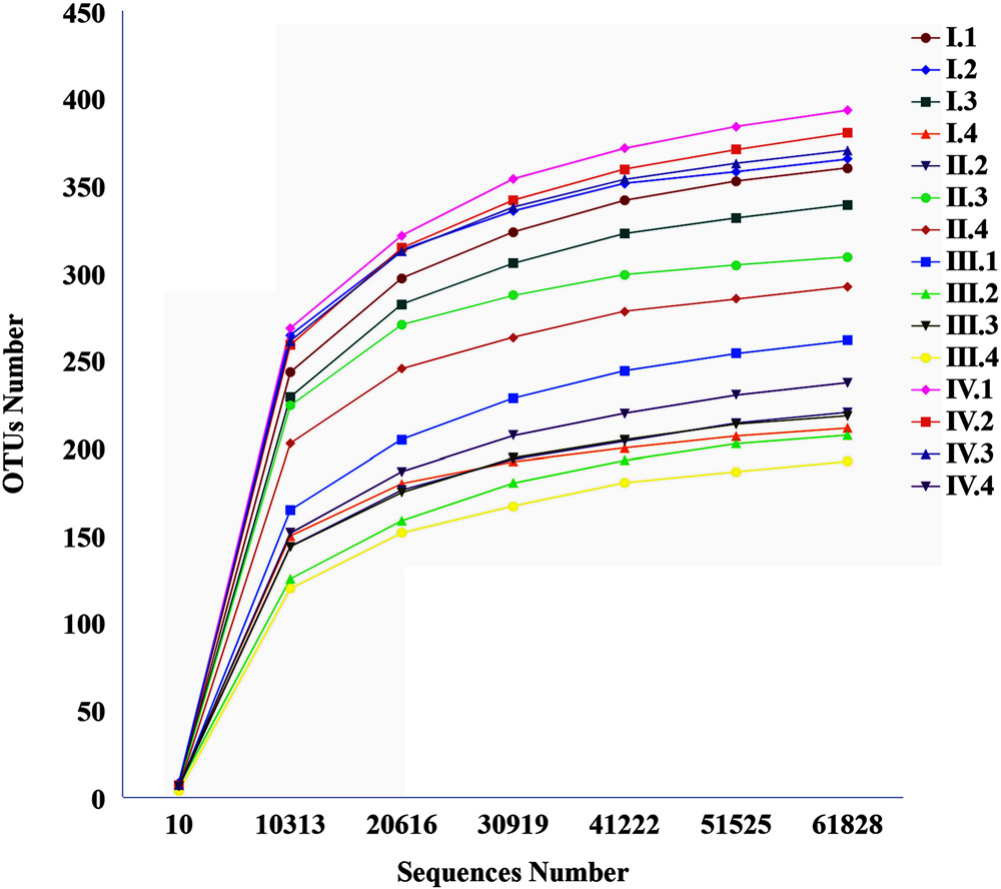
Rarefaction curve.

Extracted sequencing data and the corresponding species were used to construct a rarefaction curve (Fig. 2). As a confirmation analysis, the number of OTUs increased greatly with the increasing depth of sequencing. When the sequencing number reached 60,000, the dilution curve still had an upward trend, and it tended to flatten gradually, indicating that the amounts of sequencing data were reasonable.

### Effects of dietary NFC/NDF ratio on the alpha-diversity index of archaea.

Analysis of the alpha-diversity indices of archaea (Table 1) showed that there were some differences in the number, richness, and diversity of the species.

**TABLE 1:** Analysis of archaeal alpha diversity at a 0.03 distance in Karakul sheep^*a*^

Items	Group or parameter	No. of observed species^*b*^	Shannon	Simpson	Chao1	ACE
Period I	1	364	5.000	0.927	373.750	375.035
	2	348	4.194	0.870	361.778	361.965
	3	342	4.287	0.887	363.969	360.229
	4	218	3.762	0.847	229.040	230.354
	SEM	34.225	0.249	0.049	33.847	29.568
	*P*	0.459	0.208	0.963	0.422	0.286
						
Period II	1	344	4.351	0.853	157.800	360.647
	2	318	4.151	0.823	332.500	335.602
	3	314	4.017	0.831	326.536	326.096
	4	299	3.766	0.818	312.405	315.835
	SEM	16.203	0.267	0.019	33.180	14.458
	*P*	0.848	0.918	0.940	0.190	0.781
						
Period III	1	251	3.574	0.789	273.885	268.931
	2	216	3.456	0.822	231.241	231.374
	3	207	3.363	0.765	228.037	229.458
	4	190	2.429	0.671	204.040	204.813
	SEM	15.275	0.200	0.049	15.384	16.407
	*P*	0.611	0.148	0.781	0.502	0.648
						
Period IV	1	385	4.233	0.861	403.594	400.228
	2	379	4.482	0.899	390.234	393.561
	3	368	4.576	0.885	390.286	388.158
	4	241	3.907	0.872	271.750	269.917
	SEM	28.237	0.179	0.059	30.090	35.039
	*P*	0.227	0.617	0.998	0.412	0.562

a Periods were as follows: I (1 to 18 days), II (19 to 36 days), III (37 to 54 days), and IV (55 to 72 days). Groups 1, 2, 3, and 4 represent four groups of Karakul sheep treated with four dietary NFC/NDF ratios—0.54, 0.96, 1.37, and 1.90, respectively, here and in subsequent tables.

b Unless noted otherwise in column 2.

### Effects of dietary NFC/NDF ratio on the archaeal structure of Karakul sheep.

**FIG 3:**
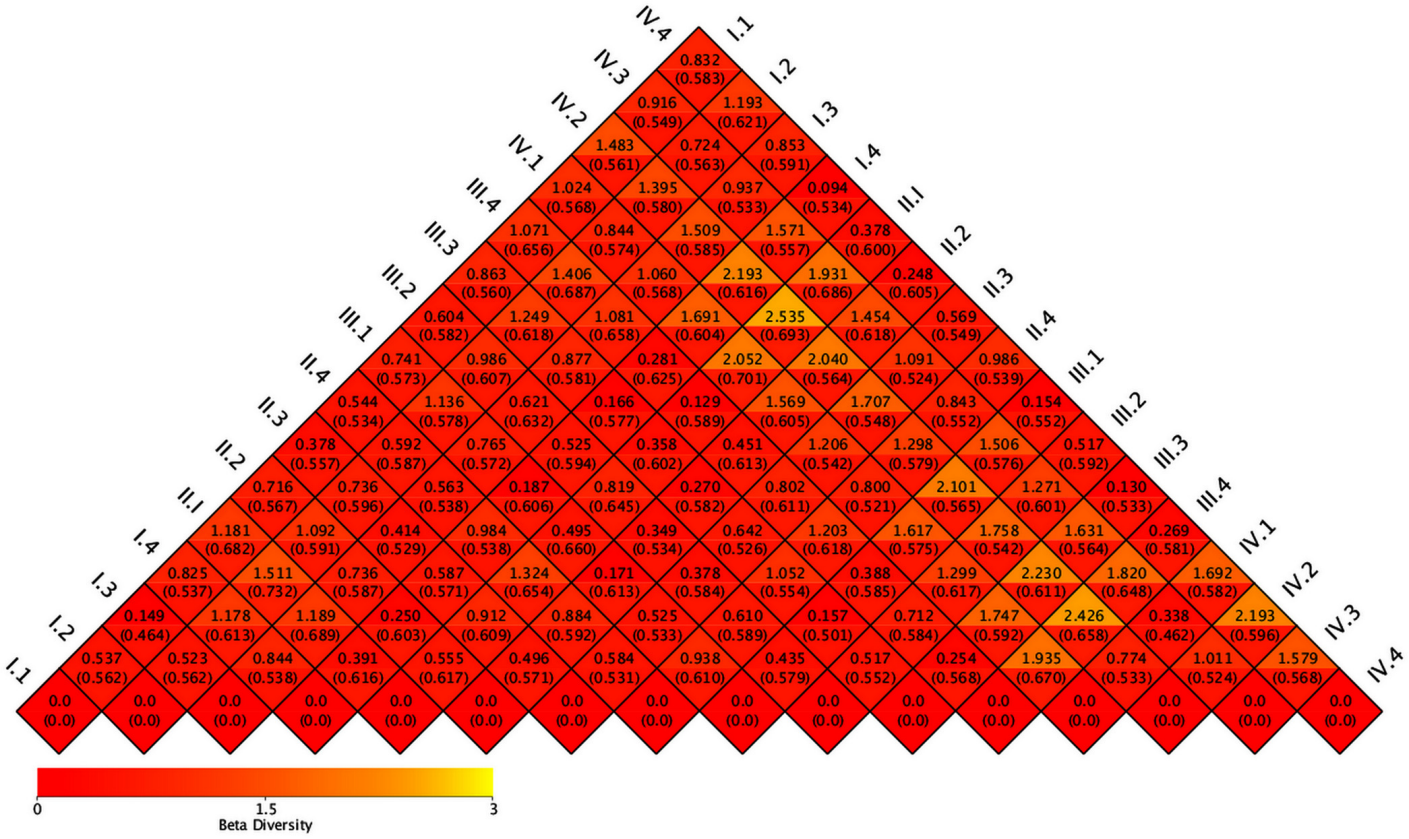
Heat map of beta diversity.

A heat map of beta-diversity index is shown in Fig. 3. In period I, the distances between group 2 and groups 1, 3, and 4 were 0.540, 0.526, and 1.185, respectively. In period II, the distances between group 2 and groups 1, 3, and 4 were 0.588, 0.493, and 0.888, respectively. In period III, the distances between group 2 and groups 1, 3, and 4 were 0.436, 0.519, and 0.714, respectively. In period IV, the distance between group 2 and groups 1, 3, and 4 were 0.773, 1.01, and 2.192, respectively. Among the four periods, the difference between group 1 and group 3 was the smallest, and the difference between group 2 and group 4 was the largest.

### Effects of dietary NFC/NDF ratio on the abundance of methanogens in Karakul sheep.

**FIG 4:**
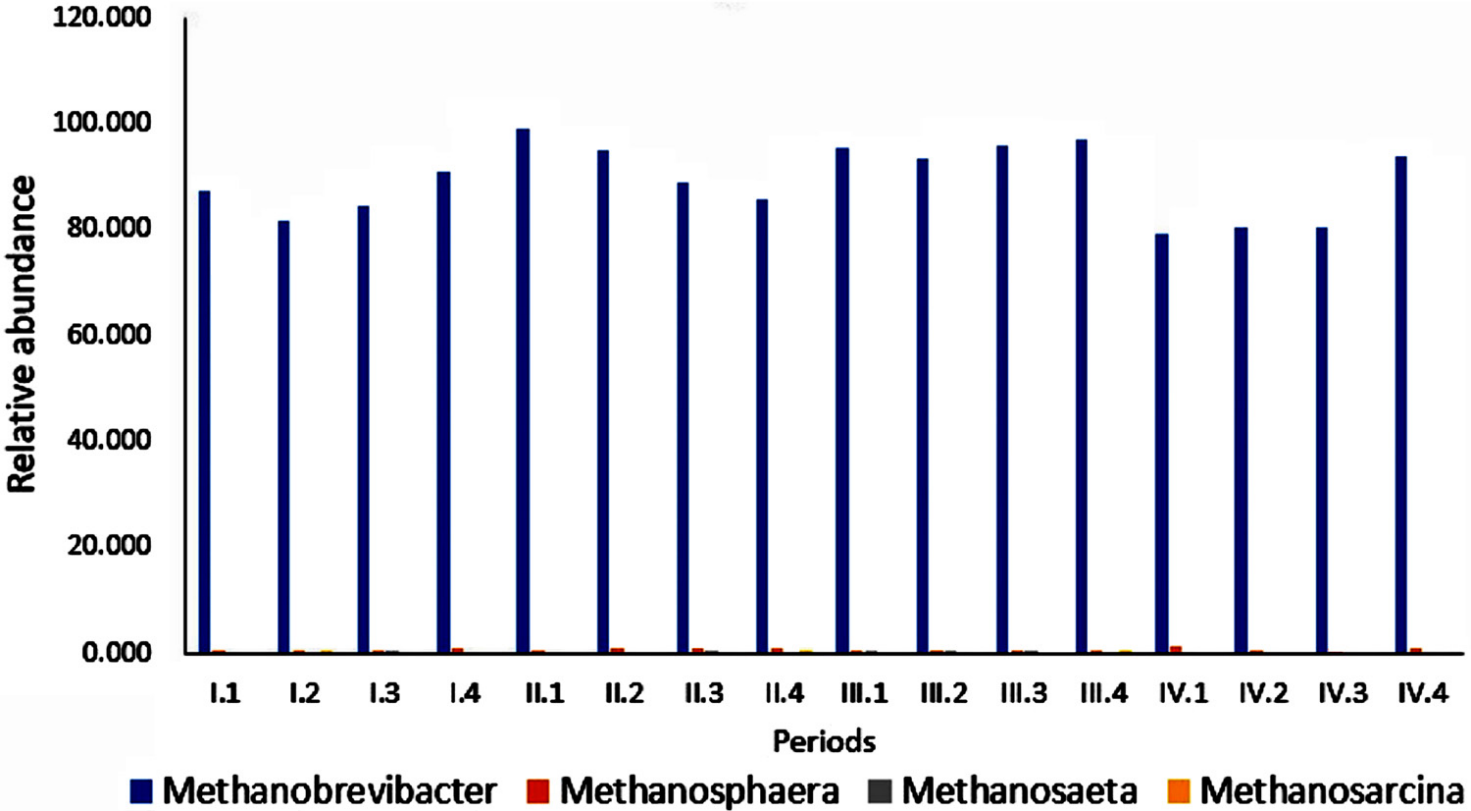
Effects of dietary NFC/NDF ratio on the relative abundance of methanogens (genus).

At the genus level, 46 genera were sequenced, and the relative abundances of the main genus among the four periods are shown in Table 2. The main methanogen genera detected were *Methanobrevibacter*, *Methanosphaera*, *Methanosaeta*, and *Methanosarcina*. The main dominant genus was *Methanobrevibacter* (79 to 99%) among the four periods. Figure 4 shows the main methanogen genera detected. The relative abundances of *Methanobrevibacter* were as follows: group 4 > group 3 > group 1 > group 2 in periods III and IV. The difference was not significant (*P* > 0.05).

**TABLE 2:** Abundance of archaea in Karakul sheep (genus)

Period and genus	Group				SEM	*P*
	1	2	3	4		
Period I						
*Methanobrevibacter*	87.416	81.741	84.570	90.838	3.539	0.866
*Methanosphaera*	0.479	0.170	0.196	0.781	0.113	0.074
*Sphaerochaeta*	0.039	0.459	0.013	−	0.080	0.053
Unidentified *Lachnospiraceae*	0.194	0.149	0.095	0.021	0.025	0.060
*Methanosaeta*	−^*a*^	−	0.273	−	0.047	0.068
*Succiniclasticum*	0.032	0.141	0.040	0.008	0.034	0.184
Unidentified-*Ruminococcaceae*	0.016	0.157	0.008	−	0.028	0.115
*Methanosarcina*	−	0.052	−	−	0.009	0.079
Unidentified-*Rickettsiales*	0.063	0.002	0.104	0.044	0.019	0.313
Period II						
*Methanobrevibacter*	98.929	95.124	88.959	85.561	3.768	0.300
*Methanosphaera*	0.265	0.704	0.956	0.851	0.103	0.211
*Sphaerochaeta*	−	0.008	−	0.002	0.001	0.068
Unidentified *Lachnospiraceae*	0.008	0.023	0.327	0.026	0.055	0.088
*Methanosaeta*	0.000	−	0.018	0.000	0.003	0.056
*Succiniclasticum*	0.003	0.032	0.018	0.013	0.005	0.245
Unidentified-*Ruminococcaceae*	0.002	0.037	−	0.099	0.018	0.149
*Methanosarcina*	−	−	−	0.113	0.020	0.094
Unidentified-*Rickettsiales*	−	0.002	−	0.026	0.004	0.059
Period III						
*Methanobrevibacter*	95.446	93.216	95.933	96.879	2.171	0.638
*Methanosphaera*	0.487	0.281	0.613	0.611	0.103	0.349
*Sphaerochaeta*	0.013	0.006	0.013	0.002	0.002	0.140
Unidentified *Lachnospiraceae*	0.058	0.018	0.087	0.015	0.013	0.719
*Methanosaeta*	0.034	0.006	0.002	−	0.006	0.093
*Succiniclasticum*	0.010	0.034	0.002	0.021	0.005	0.179
Unidentified-*Ruminococcaceae*	0.006	0.018	0.006	0.023	0.004	0.327
*Methanosarcina*	−	−	−	0.026	0.005	0.066
Unidentified-*Rickettsiales*	−	0.015	−	−	0.003	0.081
Period IV						
*Methanobrevibacter*	80.069	79.375	80.134	93.888	3.409	0.190
*Methanosphaera*	1.253	0.519	0.702	0.907	0.181	0.231
*Sphaerochaeta*	0.008	0.042	−	−	0.007	0.066
Unidentified *Lachnospiraceae*	0.097	0.039	0.107	0.082	0.016	0.535
*Methanosaeta*	−	−	−	−	−	−
*Succiniclasticum*	0.023	0.220	0.010	0.003	0.039	0.118
Unidentified-*Ruminococcaceae*	0.061	0.052	0.047	0.027	0.010	0.710
*Methanosarcina*	−	−	−	−	−	−
Unidentified-*Rickettsiales*	0.040	0.013	0.058	0.002	0.010	0.137

a -,not detected.

### Effects of dietary NFC/NDF ratio on methanogens and cellulose-degrading bacteria in Karakul sheep.

At the species level, the main methanogens and cellulose-degrading bacteria detected are shown in Table 3. The main methanogens were *Methanobrevibacter millerae*, *Methanobrevibacter ruminantium*, *Methanobrevibacter wolinii*, and *Methanosphaera* sp. strain ISO3-F5. The main cellulose-degrading bacteria detected were *Butyrivibrio fibrisolvens*, *Fibrobacter* sp. UWCM, *Ruminococcus flavefaciens*, and *Ruminococcus albus*.

**TABLE 3:** Dietary NFC/NDF ratio on methanogens and cellulose-degrading bacteria (species) of Karakul sheep

Period and species	Group^*a*^				SEM	*P*
	1	2	3	4		
Period I						
*Methanobrevibacter millerae*	14.343^B^	10.395^C^	18.343^B^	25.405^A^	1.890	<0.010
*Methanobrevibacter ruminantium*	0.428^B^	3.470^A^	0.707^B^	0.499^B^	1.890	0.030
*Methanobrevibacter wolinii*	0.312^B^	0.126^B^	0.513^B^	1.330^A^	1.580	<0.010
*Methanosphaera* sp. ISO3-F5	0.190^B^	0.143^B^	0.307^B^	0.756^A^	0.080	<0.010
*Butyrivibrio fibrisolvens*	2.664^A^	0.466^B^	2.990^A^	2.123^A^	0.371	0.035
*Fibrobacter* sp. UWCM	−	0.027	−	−	0.006	0.441
*Ruminococcus flavefaciens*	0.027	0.054	0.051	−	0.038	0.216
*Ruminococcus albus*	−	0.055	−	−	0.009	0.052
Period II						
*Methanobrevibacter millerae*	31.484^B^	7.494^C^	38.048^A^	41.195^A^	4.050	<0.010
*Methanobrevibacter ruminantium*	0.305^B^	1.878^A^	1.521^A^	0.604^B^	0.208	<0.010
*Methanobrevibacter wolinii*	0.025^C^	0.003^C^	0.329^B^	0.618^A^	0.083	<0.010
*Methanosphaera* sp. ISO3-F5	0.446^B^	0.218^C^	0.558^B^	0.873^A^	0.080	<0.010
*Butyrivibrio fibrisolvens*	2.689^A^	0.548^B^	3.123^A^	2.406^A^	1.132	0.027
*Fibrobacter* sp. UWCM	0.082	0.411	0.027	−	0.350	0.294
*Ruminococcus flavefaciens*	−	0.055	0.050	0.027	0.036	0.290
*Ruminococcus albus*	0.027	0.055	−	−	0.010	0.561
Period III						
*Methanobrevibacter millerae*	35.036^A^	11.464^C^	42.022^A^	46.415^A^	4.080	<0.010
*Methanobrevibacter ruminantium*	0.345^B^	1.234^A^	0.417^B^	0.381^B^	0.128	<0.010
*Methanobrevibacter wolinii*	0.005^C^	−	0.074^B^	0.163^A^	0.020	<0.010
*Methanosphaera* sp. ISO3-F5	0.386^C^	0.185^C^	0.530^B^	0.855^A^	0.056	<0.010
*Butyrivibrio fibrisolvens*	6.785^A^	0.657^C^	7.687^A^	3.292^B^	1.143	<0.010
*Fibrobacter* sp. UWCM	0.025	0.027	−	−	0.009	0.596
*Ruminococcus flavefaciens*	0.027	0.082	0.055	0.027	0.021	0.627
*Ruminococcus albus*	−	0.093	−	−	0.023	0.073
Period IV						
*Methanobrevibacter millerae*	23.601^B^	13.346^C^	24.425^AB^	29.986^A^	1.966	<0.010
*Methanobrevibacter ruminantium*	1.347^B^	2.567^A^	2.113^A^	1.647^B^	1.760	<0.010
*Methanobrevibacter wolinii*	0.004^C^	0.002^C^	0.147^B^	0.214^A^	0.030	<0.010
*Methanosphaera* sp. ISO3-F5	0.470^B^	0.348^C^	0.758^AB^	0.899^A^	0.080	0.030
*Butyrivibrio fibrisolvens*	7.234^A^	2.301^C^	8.694^A^	5.975^B^	1.581	<0.010
*Fibrobacter* sp. UWCM	−	0.082	−	−	0.070	0.441
*Ruminococcus flavefaciens*	−	0.082	0.079	−	0.016	0.052
*Ruminococcus albus*	−	0.085	−	−	0.013	0.063

a In the same row, values with no letter or the same letter superscripts mean no significant difference (*P* > 0.05), while with different letter superscripts mean significant difference (*P* < 0.05). -, not detected.

Among the four periods, the relative abundance of methanogens was lowest in group 2 and highest in group 4. The relative abundances of *Methanobrevibacter millerae*, *Methanobrevibacter wolinii*, and *Methanosphaera* sp. ISO3-F5 were as follows: group 4 > group 3 > group 1 > group 2. The difference was significant (*P < *0.05). The relative abundance of *Methanobrevibacter ruminantium* was as follows: group 2 > group 3 > group 4 > group 1. The difference was significant (*P < *0.05).

Among the four periods, the relative abundance of cellulose-degrading bacteria was lowest in group 2 and highest in group 3. The relative abundance of *Butyrivibrio fibrisolvens* was highest and was as follows: group 3 > group 1 > group 4 > group 2. The difference was significant (*P < *0.05). The relative abundances of *Fibrobacter* sp. UWCM, *Ruminococcus flavefaciens*, and *Ruminococcus albus* were reached highest in group 2.

These results show that when the dietary NFC/NDF ratio was 0.96, the relative abundances of methanogens and cellulose-degrading bacteria were the lowest. When the NFC/NDF ratio in the diet was >0.96, the relative abundance of methanogens increased, while cellulose-degrading bacteria first increased and then decreased.

## DISCUSSION

### Effects of dietary NFC/NDF ratio on methanogens in Karakul sheep.

A recently published comprehensive review highlighted the needs to study the type of carbohydrate impact on methane production (21). Rumen archaea account for 0.3 to 3.3% of rumen microorganisms (22), and these are the only microorganisms in the rumen that can produce CH_4_ (23, 24). So far, 108 species of methanogens have been collected by the U.S. National Center for Biotechnology Information. The main representative species are Methanobrevibacter ruminantium, Methanomicrobium mobile, Methanobacterium formicicum, Methanosarcinaceae barkeri, and Methanobrevibacter smithii. Many studies have shown that the main methanogen in ruminants is *Methanobrevibacter* (25, 26). Mao et al. (27) showed that the structure of methanogens did not change when the proportion of dietary concentrate increased from 25 to 50%. Kumar et al. (28) showed that when the proportion of concentrate in dairy cows increased from 25 to 50%, there was little effect on the structure of methanogen, and *Brevibacter* was the dominant methanogen, accounting for 96%. These results may be due to a low methanogen content in ruminants, to the structure of methanogen being stable when the animals are very young, or to a difference in sequencing methods (29). Shuo et al. (30) found that the quantity of methanogens (1.68 × 10^8^/mL) in the feeding group was significantly lower than in the natural grazing group (1.22 × 10^9^/mL), but the dominant methanogen did not change. Hook et al. (31) showed that a high-concentrate diet had little effect on the density of methanogenic bacteria but significantly affected the diversity of methanogenic bacteria. In this experiment, the main dominant methanogen was *Methanobrevibacter*, which is consistent with the findings of Kim et al. (32), but the diversity of archaea decreased with an increase in the dietary NFC/NDF ratio. Recent research has shown that CH_4_ production decreases with an increase in feed concentrate (33, 34). Zhou et al. (35) found that a difference in feed efficiency may cause the methanogenic bacterial community difference, and Sha et al. (36) suggested that the methane emission level was closely related to the methanogen content. Furthermore, in this experiment, the relative abundance of methanogens was lowest at a dietary NFC/NDF ratio of 0.96 and gradually increased when the dietary NFC/NDF ratio was >0.96 among the four periods. This may be because the increase in rapidly digestible nutrients provided more energy for the methanogens.

### Effects of dietary NFC/NDF ratio on cellulose-degrading bacteria in Karakul sheep.

Improving fiber degradation rate is very important for ruminants. Bacteria and fungi play an important role in the decomposition and utilization of cellulose, especially with cellulose-degrading bacteria such as Ruminococcus flavefaciens, Fibrobacter succinogenes, Butyrivibrio fibrisolvens, and *Clostridium* spp. Research has shown that diets supplemented with readily fermentable carbohydrates could decrease rumen fiber degradation (37). Chen et al. (38) indicated that the abundance of cellulose-degrading bacteria was closely related to the cellulose-degrading bacterial effects on gut microbiota. In this experiment, the relative abundance of total cellulose-degrading bacteria was highest when the dietary NFC/NDF ratio was 1.37, and it decreased when the NFC/NDF ratio was >1.37. These results were consistent with the results obtained by our lab previously. The apparent ADF and NDF degradation rates were highest when the dietary NFC/NDF ratio was 1.37; this may be related to the relative abundance of cellulose-degrading bacteria in the rumen (39). Sha et al. (35) showed that a higher content of cellulose-degrading bacteria in the rumen indicates a greater capacity to digest fibrous material, which translates into higher production efficiency. Chen et al. (38) also confirmed that cellulose-degrading bacteria in the rumen promoted the degradation of cellulose. The relative abundance of *Fibrobacter* sp. UWCM, *Ruminococcus flavefaciens*, and *Ruminococcus albus* was low, which is consistent with the findings of Zened et al. (40). Research has shown that the *Ruminococcus flavefaciens* content was 100-fold greater than the *Ruminococcus albus* content in the rumen (41), but in our research *Ruminococcus albus* detected was similar to *Ruminococcus flavefaciens*; this discrepancy might be due to the species difference and the sequencing method. Research has also shown that high-energy diets affected the activity of the cellulose enzyme more than the number of cellulose-degrading bacteria (42). This needs to be further investigated in future experiments.

### Conclusions.

The diversity of archaea decreased with an increase in the dietary NFC/NDF ratio. When the NFC/NDF ratio was 0.96, it had a great influence on the structure of archaea, and the relative abundances of methanogens and cellulose-degrading bacteria were lowest, whereas the relative abundances of total cellulose-degrading bacteria were highest when the NFC/NDF ratio was 1.37. Based on these data, we conclude that a dietary NFC/NDF ratio of 0.96 to 1.37 is appropriate for supporting optimal sheep production.

## MATERIALS AND METHODS

All experimental procedures were approved by Tarim University Animal Care and Use Committee, and humane animal care procedures were followed throughout the experiment.

### Animals and dietary composition.

Twelve male Karakul sheep weighed (35 ± 3.3 kg) with fistulas were randomly divided into four groups, each with three replicates, and were fed with four dietary NFC/NDF ratios of 0.54, 0.96, 1.37, and 1.90, respectively, as groups 1, 2, 3, and 4. The NFC/NDF ratio design of the four diets was based on previously reported ration concentrate/roughage ratios (43–45), which can meet the growth needs of sheep and are considered the common ration offered to local Karakul sheep. The concentrate/roughage ratios were 37:63, 50:50, 60:40, and 63:37, respectively. All sheep were housed individually in metabolic cages (1.2 m × 1.5 m) and fed the experimental diet individually twice a day at 9:00 a.m. and 8:00 p.m. with free access to water. The dietary NFC/NDF ratios were formulated to meet the standards for raising meat and sheep in the People’s Republic of China (NY/T816-2004 [46]). The ingredients and nutrient levels for the diets are shown in Table 4.

**TABLE 4:** Ingredients and nutrient level of the diet

Ingredient or nutrient level	Dietary period (% dry matter)^*a*^			
	I	II	III	IV
Ingredients				
Corn	20.00	35.40	45.00	54.00
Bean pulp	2.00	2.00	2.00	2.00
Wheat bran	12.70	10.30	10.70	4.70
NaCl	0.80	0.80	0.80	0.80
CaCO_3_	0.50	0.50	0.50	0.50
Premixer^*b*^	1.00	1.00	1.00	1.00
Cotton seed hulls	30.00	20.00	15.00	13.00
Alfalfa pellets	33.00	30.00	25.00	24.00
Total	100	100	100	100
Nutrient level^*c*^				
Dry matter	95.54	95.74	96.30	95.55
Crude protein	14.76	14.96	14.10	13.43
Ether extract	2.08	2.11	2.33	2.58
Ca	0.74	0.73	0.72	0.75
P	0.26	0.25	0.24	0.22
NFC^*d*^	26.11	36.98	44.41	50.84
NDF	48.20	38.33	32.30	26.80
NFC/NDF ratio	0.54	0.96	1.37	1.90

a The experiment lasted for four periods: I (1 to 18 days), II (19 to 36 days), III (37 to 54 days), and IV (55 to 72 days).

b The premixer provided the following (per kg) diets: vitamin A, 1,800 IU; vitamin D_3_, 600 IU; vitamin E, 30 mg; Fe, 65 mg; Se, 0.15 mg; I, 0.6 mg; Cu, 10 mg; Mn, 28 mg; Zn, 45 mg; and Cu, 12 mg.

c Nutritional level was a calculated value.

d NFC = (1 − NDF − CP − Fat − Ash) × 100%.

### Experimental design and sample collection.

The experiment lasted 72 days and included four periods: period I (1 to 18 days), II (19 to 36 days), III (37 to 54 days), and IV (55 to 72 days). Each period lasted 18 days, the first 15 days was used for adaption; then, the ruminal digesta was sampled consecutively before morning feeding for 3 days. The rumen contents were collected from three sheep in one group, mixed into a 50-mL plastic container, and stored at 280°C prior to DNA extraction.

### DNA extraction, PCR, and sequencing.

The total genetic DNA was extracted from the samples using QIAamp Fast DNA Stool minikit (Tiangen, Shanghai, China) according to the manufacturer, and production was detected by using a 1% agarose gel. The extracted DNA was subjected to PCR amplification. Archaea were amplified according to the V8 region, and the specific primers used were 1106F (TTWAGTCAGGCAACGAGC) and 1378R (TGTGCAAGGAGCAGGGAC). PCR was carried out in triplicate 50-μL reactions that contained 15 μL of 2× Phusion Master Mix, 3 μL of primer, 10 μL of gDNA, and 2 μL of H_2_O. The reaction procedure consisted of predenaturation at 98°C for 1 min, followed by 30 cycles of denaturation at 98°C for 10 s, annealing at 50°C for 30 s, and extension at 72°C for 30 s, with a final extension at 72°C for 5 min.

Bacteria were amplified according to the V1-V9 region. The specific primers used were 5′-AGAGTTTGATCCTGGCTCAG-3′ (forward) and 5′-GNTACCTTGTTACGACTT-3′ (reverse). PCR was carried out in triplicate 50-μL reaction mixtures containing 2 μL of primer mix (1 μM), 5 ng of gDNA, 1 μL of Trans-FastPFU, 10 μL of 5× buffer, 5 μL of 5× StimuLate, 5 μL of deoxynucleoside triphosphates (2.5 mM concentrations of each), and 27 μL of NFW. The reaction procedure consisted of predenaturation at 95°C for 2 min, followed by 35 cycles of denaturation at 95°C for 30 s, annealing at 60°C for 45 s, and extension at 72°C for 90 s, with a final extension at 72°C for 10 min.

The specific primers with barcodes were synthesized by Biological Engineering Co., Ltd., and amplified PCR results were detected by using a 2% agarose gel. Archaea and bacteria were purified by using an adhesive recovery kit. Finally, archaea were sequenced on an Ion S5TMXL platform, and bacteria were sequences on a PacBio platform.

### Data analysis.

First raw data were processed into clean reads. Then, Uparse software (47) was used to cluster clean reads for all samples. OTUs were formed at a similarity of 97% (48) and annotated by using Mothur and SILVA (49) according to the reference taxonomy provided by the SSU rRNA database (50). The diversity of the OTUs was analyzed by using the QIIME pipeline. The structural diversity of archaea was analyzed by using the beta-diversity index. (This was expressed as a digital heat map that was measured by the weighted UniFrac distance.) The alpha diversity (analyzing the diversity of the microbiome) involves the Chao1 (51), ACE (52), and Shannon and Simpson (53) indices. The differences between methanogens and cellulose-degrading bacteria were compared by using the Duncan method with SPSS 17.0 software; results are presented as means, and significance was declared at *P < *0.05.

### Analysis of archaeal OTU alpha diversity.

Before alpha-diversity analysis, a rarefaction curve was determined to measure whether the data acquired were sufficient for further analysis.

### Data availability.

All data generated during this study are included in this published article and in its supplemental material files.
