# Supplementary material for: Effects of Dietary Nonfibrous Carbohydrate/Neutral Detergent Fiber Ratio on Methanogenic Archaea and Cellulose-Degrading Bacteria in the Rumen of Karakul Sheep: a 16S rRNA Gene Sequencing Study
Source: Appl Environ Microbiol. 2022 Dec 21;89(1):e01291-22. doi: 10.1128/aem.01291-22 (PMC9888294; doi:10.1128/aem.01291-22)
Supplement: Supplemental file 1 — Supplemental material. Download aem.01291-22-s0001.pdf, PDF file, 0.7 MB [file aem.01291-22-s0001.pdf]

Supplemental Material

Effects of Dietary Non-fibrous Carbohydrate/Neutral Detergent Fiber Ratio on Methanogenic Archaea and Cellulose-degrading Bacteria in the Rumen of Karakul Sheep-a 16S rDNA Sequencing Study

Running title: Dietary Non-fibrous Carbohydrate/Neutral Detergent Fiber Ratio on Methanogenic Archaea and Cellulose-degrading Bacteria.

Tiantian Bai[#a], Xuanxuan Pu[#a], Xuefeng Guo[*a,b], Junfeng Liu[*a,b], Linbo Zhao[a], Xiuping Zhang[a,b], Sujiang Zhang[a,b], Long Cheng[c]

[a] College of Animal Science and Technology, Tarim University, Alar 843300, Xinjiang, PR China; [b] Key Laboratory of Tarim Animal Husbandry Science and Technology of Xinjiang Production and Construction Group, Alar 843300, Xinjiang, PR China; [c] Faculty of Veterinary & Agricultural Sciences, Dookie College, The University of Melbourne, Victoria 3647, Australia

#Contributed equally.

*Address correspondence to Xuefeng Guo, gxfdky@126.com and Junfeng Liu, ljfdky@126.com.

Tiantian Bai and Xuanxuan Pu contributed equally to this paper. Author order was determined by the writing of the paper and the division of labor for conducting experiments.

Table S1 Analysis of archaea Alpha diversity at 0.03 distance in Karakul sheep

Items	Group	Observed-species	Shannon	Simpson	Chao1	ACE
	1	364	5.000	0.927	373.750	375.035
Period I	2	348	4.194	0.870	361.778	361.965
	3	342	4.287	0.887	363.969	360.229
	4	218	3.762	0.847	229.040	230.354
	SEM	34.225	0.249	0.049	33.847	29.568
	P-value	0.459	0.208	0.963	0.422	0.286
	1	344	4.351	0.853	157.800	360.647
Period II	2	318	4.151	0.823	332.500	335.602
	3	314	4.017	0.831	326.536	326.096
	4	299	3.766	0.818	312.405	315.835
	SEM	16.203	0.267	0.019	33.180	14.458
	P-value	0.848	0.918	0.940	0.190	0.781
	1	251	3.574	0.789	273.885	268.931
Period III	2	216	3.456	0.822	231.241	231.374
	3	207	3.363	0.765	228.037	229.458
	4	190	2.429	0.671	204.040	204.813
	SEM	15.275	0.200	0.049	15.384	16.407
	P-value	0.611	0.148	0.781	0.502	0.648
	1	385	4.233	0.861	403.594	400.228
Period IV	2	379	4.482	0.899	390.234	393.561

	3	368	4.576	0.885	390.286	388.158
	4	241	3.907	0.872	271.750	269.917
	SEM	28.237	0.179	0.059	30.090	35.039
	P-value	0.227	0.617	0.998	0.412	0.562

Note: Period I (1~18 d), II (19~36 d), III (37~54 d) and IV (55~72 d) and Group 1, 2, 3, 4 means four groups of Karakul sheep treated with four dietary NFC/NDF ratios of 0.54、0.96、1.37 and 1.90, respectively. The same as below.

Table S2 Abundance of archaea in Karakul sheep (genus)

	Period I					
Genus	1	2	3	4	SEM	*P*-value
Methanobrevibacter	87.416	81.741	84.570	90.838	3.539	0.866
Methanosphaera	0.479	0.170	0.196	0.781	0.113	0.074
Sphaerochaeta	0.039	0.459	0.013	—	0.080	0.053
Unidentified-Lachnospiraceae	0.194	0.149	0.095	0.021	0.025	0.060
Methanosaeta	—	—	0.273	—	0.047	0.068
Succiniclasticum	0.032	0.141	0.040	0.008	0.034	0.184
Unidentified-Ruminococcaceae	0.016	0.157	0.008	—	0.028	0.115
Methanosarcina	—	0.052	—	—	0.009	0.079
Unidentified-Rickettsiales	0.063	0.002	0.104	0.044	0.019	0.313

	Period II					
Genus	1	2	3	4	SEM	*P*-value
Methanobrevibacter	98.929	95.124	88.959	85.561	3.768	0.300
Methanosphaera	0.265	0.704	0.956	0.851	0.103	0.211
Sphaerochaeta	—	0.008	-	0.002	0.001	0.068
Unidentified-Lachnospiraceae	0.008	0.023	0.327	0.026	0.055	0.088

Methanosaeta	0.000	—	0.018	0.000	0.003	0.056
Succiniclasticum	0.003	0.032	0.018	0.013	0.005	0.245
Unidentified-Ruminococcaceae	0.002	0.037	—	0.099	0.018	0.149
Methanosarcina	—	—	—	0.113	0.020	0.094
Unidentified-Rickettsiales	—	0.002	—	0.026	0.004	0.059

	Period III					
Genus	1	2	3	4	SEM	*P*-value
Methanobrevibacter	95.446	93.216	95.933	96.879	2.171	0.638
Methanosphaera	0.487	0.281	0.613	0.611	0.103	0.349
Sphaerochaeta	0.013	0.006	0.013	0.002	0.002	0.140
Unidentified-Lachnospiraceae	0.058	0.018	0.087	0.015	0.013	0.719
Methanosaeta	0.034	0.006	0.002	—	0.006	0.093
Succiniclasticum	0.010	0.034	0.002	0.021	0.005	0.179
Unidentified-Ruminococcaceae	0.006	0.018	0.006	0.023	0.004	0.327
Methanosarcina	—	—	—	0.026	0.005	0.066
Unidentified-Rickettsiales	—	0.015	—	—	0.003	0.081

	Period IV					
Genus	1	2	3	4	SEM	*P*-value
Methanobrevibacter	80.069	79.375	80.134	93.888	3.409	0.190
Methanosphaera	1.253	0.519	0.702	0.907	0.181	0.231
Sphaerochaeta	0.008	0.042	—	—	0.007	0.066
Unidentified-Lachnospiraceae	0.097	0.039	0.107	0.082	0.016	0.535
Methanosaeta	—	—	—	—	—	—
Succiniclasticum	0.023	0.220	0.010	0.003	0.039	0.118
Unidentified-Ruminococcaceae	0.061	0.052	0.047	0.027	0.010	0.710
Methanosarcina	—	—	—	—	—	—
Unidentified-Rickettsiales	0.040	0.013	0.058	0.002	0.010	0.137

Table S3 Dietary NFC/NDF ratio on methanogens and cellulose-degrading bacteria (species) of Karakul sheep

	Period I					
Species	1	2	3	4	SEM	*P*-value
Methanobrevibacter-millerae	14.343[b]	10.395[c]	18.343[b]	25.405[a]	1.890	<0.010
Methanobrevibacter-ruminantium	0.428[b]	3.470[a]	0.707[b]	0.499[b]	1.890	0.030
Methanobrevibacter-wolinii	0.312[b]	0.126[b]	0.513[b]	1.330[a]	1.580	<0.010
Methanosphaera-sp-ISO3-F5	0.190[b]	0.143[b]	0.307[b]	0.756[a]	0.080	<0.010
Butyrivibrio-fibrisolvens	2.664[a]	0.466[b]	2.990[a]	2.123[a]	0.371	0.035
Fibrobacter-sp-UWCM	—	0.027	—	—	0.006	0.441
Ruminococcus-flavefaciens	0.027	0.054	0.051	—	0.038	0.216
Ruminococcus-albus	—	0.055	—	—	0.009	0.052

	Period II					
Species	1	2	3	4	SEM	*P*-value

Methanobrevibacter-millerae	31.484^{b}	7.494^{c}	38.048^{a}	41.195^{a}	4.050	<0.010
Methanobrevibacter-ruminantium	0.305^{b}	1.878^{a}	1.521^{a}	0.604^{b}	0.208	<0.010
Methanobrevibacter-wolinii	0.025^{c}	0.003^{c}	0.329^{b}	0.618^{a}	0.083	<0.010
Methanosphaera-sp-ISO3-F5	0.446^{b}	0.218^{c}	0.558^{b}	0.873^{a}	0.080	<0.010
Butyrivibrio-fibrisolvens	2.689^{a}	0.548^{b}	3.123^{a}	2.406^{a}	1.132	0.027
Fibrobacter-sp-UWCM	0.082	0.411	0.027	—	0.350	0.294
Ruminococcus-flavefaciens	—	0.055	0.050	0.027	0.036	0.290
Ruminococcus-albus	0.027	0.055	—	—	0.010	0.561

	Period III					
Species	1	2	3	4	SEM	*P*-value
Methanobrevibacter-millerae	35.036^{a}	11.464^{c}	42.022^{a}	46.415^{a}	4.080	<0.010
Methanobrevibacter-ruminantium	0.345^{b}	1.234^{a}	0.417^{b}	0.381^{b}	0.128	<0.010

Methanobrevibacter-wolinii	0.005[c]	—	0.074[b]	0.163[a]	0.020	<0.010
Methanosphaera-sp-ISO3-F5	0.386[c]	0.185[c]	0.530[b]	0.855[a]	0.056	<0.010
Butyrivibrio-fibrisolvens	6.785[a]	0.657[c]	7.687[a]	3.292[b]	1.143	<0.010
Fibrobacter-sp-UWCM	0.025	0.027	—	—	0.009	0.596
Ruminococcus-flavefaciens	0.027	0.082	0.055	0.027	0.021	0.627
Ruminococcus-albus	—	0.093	—	—	0.023	0.073

	Period IV					
Species	1	2	3	4	SEM	*P*-value
Methanobrevibacter-millerae	23.601[b]	13.346[c]	24.425[ab]	29.986[a]	1.966	<0.010
Methanobrevibacter-ruminantium	1.347[b]	2.567[a]	2.113[a]	1.647[b]	1.760	<0.010
Methanobrevibacter-wolinii	0.004[c]	0.002[c]	0.147[b]	0.214[a]	0.030	<0.010
Methanosphaera-sp-ISO3-F5	0.470[b]	0.348[c]	0.758[ab]	0.899[a]	0.080	0.030

Butyrivibrio-fibrisolvens	7.234[a]	2.301[c]	8.694[a]	5.975[b]	1.581	<0.010
Fibrobacter-sp-UWCM	—	0.082	—	—	0.070	0.441
Ruminococcus-flavefaciens	—	0.082	0.079	—	0.016	0.052
Ruminococcus-albus	—	0.085	—	—	0.013	0.063

Table S4 The ingredients and nutrient composition of the diet (% of dry matter))

	I	II	III	IV
Ingredients				
Corn	20.00	35.40	45.00	54.00
Bean pulp	2.00	2.00	2.00	2.00
Wheat bran	12.70	10.30	10.70	4.70
NaCl	0.80	0.80	0.80	0.80
$CaCO_3$	0.50	0.50	0.50	0.50
Premixer①	1.00	1.00	1.00	1.00
Cotton seed hulls	30.00	20.00	15.00	13.00
Alfalfa pellets	33.00	30.00	25.00	24.00
Total	100	100	100	100
Nutritional level②				
Dry matter	95.54	95.74	96.30	95.55
Crude protein	14.76	14.96	14.10	13.43
Ether extract	2.08	2.11	2.33	2.58
Ca	0.74	0.73	0.72	0.75
P	0.26	0.25	0.24	0.22
NFC③	26.11	36.98	44.41	50.84
NDF	48.20	38.33	32.30	26.80
NFC/NDF ratio	0.54	0.96	1.37	1.90

①The premix provided the following per kg of diets: VA 1 800 IU, VD_3 600 IU, VE 30 mg, Fe

65 mg, Se 0.15 mg, I 0.6 mg, Cu 10 mg, Mn 28 mg, Zn 45 mg, Cu 12 mg.

②Nutritional level was a calculated value.

③NFC=（1−NDF−CP−Fat−Ash）× 100%.

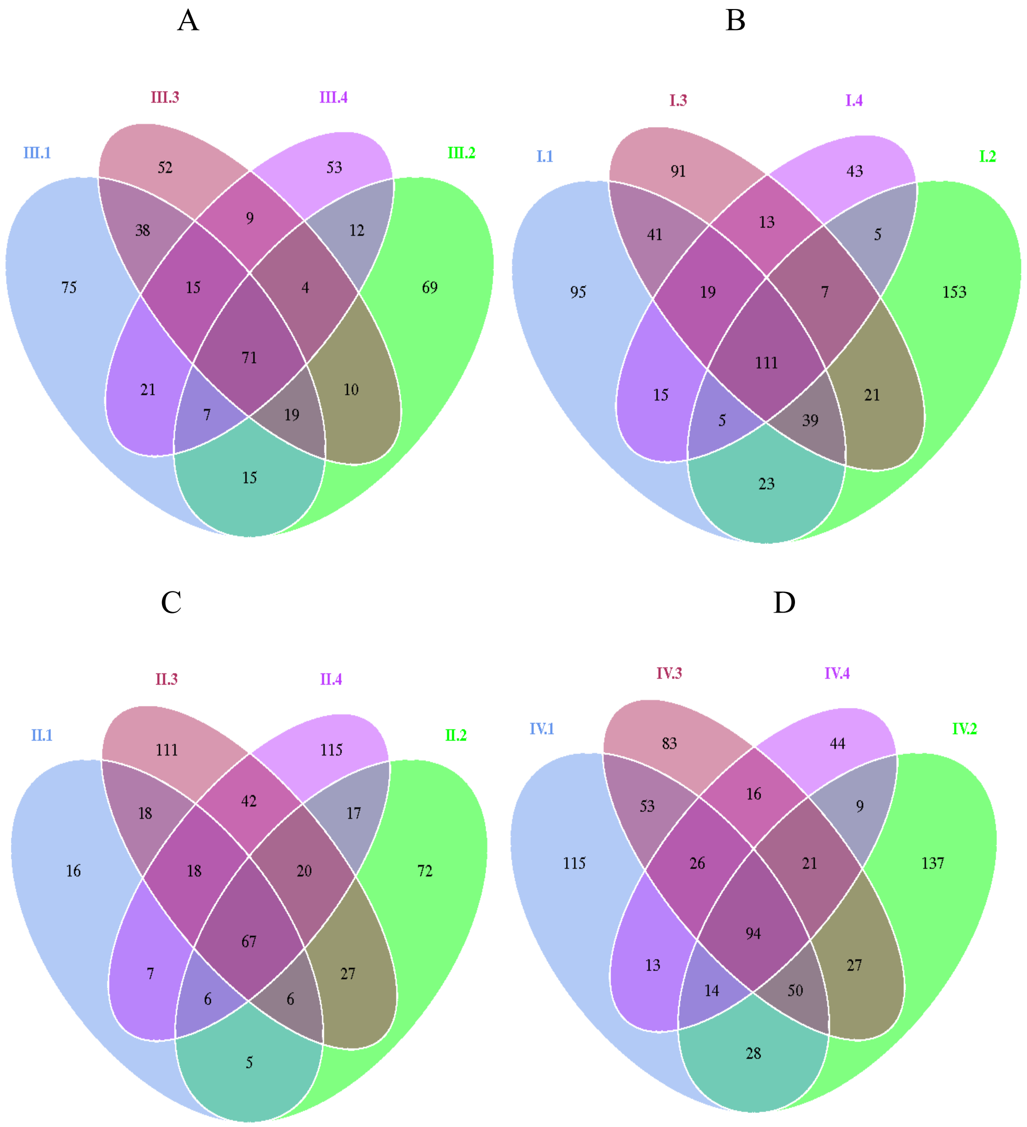

Figure S1 Venn graph of archaea OTUs in Karakul sheep. A, B, C and D represents Period I, Period II, Period III and Period IV, respectively. Period I (1~18 d), II (19~36 d), III (37~54 d) and IV (55~72 d) and Group 1, 2, 3, 4 means four groups of Karakul sheep treated with four dietary NFC/NDF ratios of 0.54、0.96、1.37 and 1.90 respectively. The same color represents the same OTUs among each group, while different colors represent the unique OTUs of each group in the figure. The same as below.

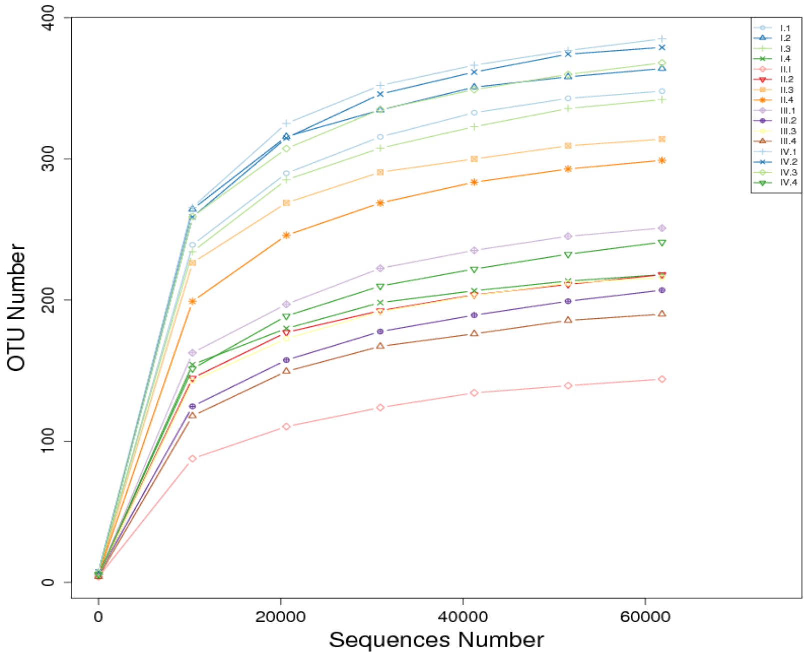

Figure S2 Rarefaction curve. the number of OTUs increased greatly with the increase of sequencing depth, indicating that the amount of sequencing data was reasonable.

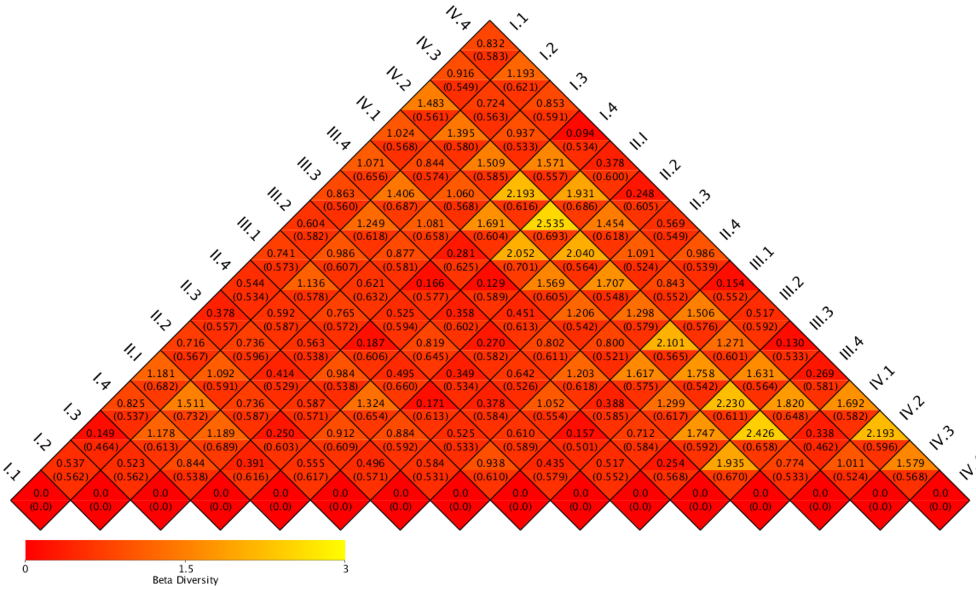

Figure S3 Heat map of Beta diversity

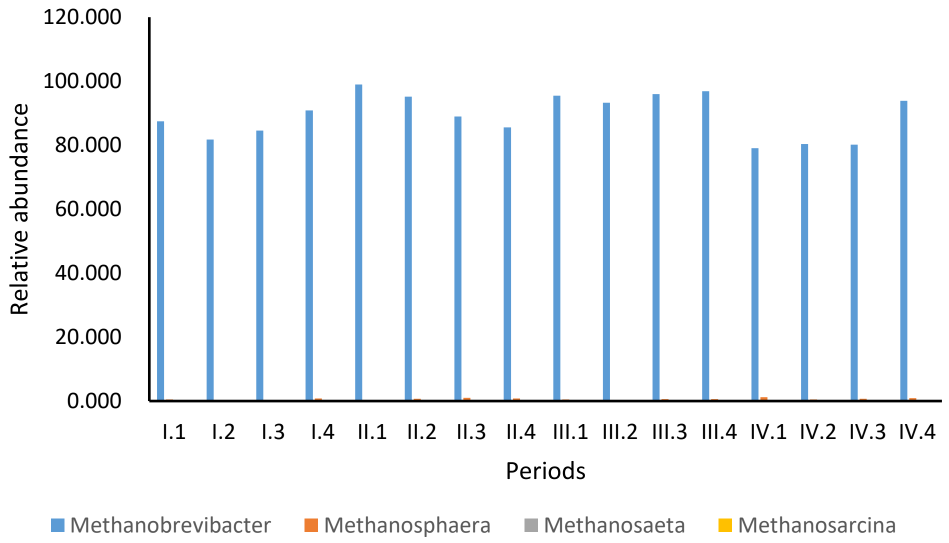

Figure S4 Effects of dietary NFC/NDF ratio on the relative abundance of methanogens (genus)
